# Prediction of textile pilling resistance using optical coherence tomography

**DOI:** 10.1038/s41598-022-23230-9

**Published:** 2022-10-31

**Authors:** Jarosław Gocławski, Joanna Sekulska-Nalewajko, Ewa Korzeniewska

**Affiliations:** 1 Institute of Applied Computer Science, Lodz University of Technology, Stefanowskiego 18, 90-537 Lodz, Poland; 2 Institute of Electrical Engineering Systems, Lodz University of Technology, Stefanowskiego 18, 90-537 Lodz, Poland

**Keywords:** Imaging techniques, Surface patterning, Imaging and sensing, Software, Information technology, Computational methods

## Abstract

This paper describes a new method of textile pilling prediction, based on multivariate analysis of the spatial layer above the surface. The original idea of the method is the acquisition of 3D fabric image using optical coherence tomography (OCT) with infrared light, which allows for the fabric fuzz visualization with high sensitivity. The pilling layer, reconstructed with the resolution of $10\times 10\times 5.5 \; \upmu \mathrm {m}$, includes reliable textural information related to the amount of loose fibers and bunches appearing as a result of abrasion. Pilling intensity was assigned by supervised classification of the textural features using both linear (PLS-DA - partial least squares discriminant analysis, LDA - linear discriminant analysis) and non-linear (SVM - support vector machine) classifiers. The results demonstrated that the method is more suitable for fabrics after short-term abrasion, when the fuzz prevails over tangled fibers in the pilling layer. In that case, pilling grades were predicted with $>98\%$ accuracy, sensitivity and specificity (for SVM model). The validation accuracy of the tested models after machine abrasion achieves lower values (up to $90.4\%$ for LDA model). With our method, we clearly showed that OCT can be used to quantitatively trace appearance changes of fabric samples due to test abrasion.

## Introduction

Pilling is a fabric defect that appears on the textile surface as a result of repeated abrasions when washing or wearing clothes. Undesirable pills of various sizes attached to the surface of the fabric are the main symptom of this phenomenon. The clearly visible pills and the resulting fluff affect both the value of the garment and the comfort of its use. Pilling is associated with intensive use, the density of the fabric weave, and specific characteristics of the fibers, such as high hairiness^1^. Technical standards provide techniques and principles for testing fabrics for pilling (e.g. ISO-12945-2: 2021-04). To assess their resistance to pilling, fabrics are subjected to surface friction. Wear tests include the random tumble method, the accelerated wear test, Hartr’s pilling test, the appearance retention test, the brush sponge type wear test, the inflatable mode wear test, and the Martindale pilling method, which is the most popular.

The pilling tendency of a fabric is usually assessed visually by a panel of experts on a five-point scale. The sample is first subjected to one of the pilling methods and then compared with photographic standards. Examples of existing standards include the EMPA standard (EMPA – Eidgenössische Materialprüfungs- und ForschungsAnstalt, Swiss Federal Laboratories for Materials Testing and Research), which is used with the Martindale test, and ASTM Photographic Standards, which are used with the Random Tumble Pilling Tester. Existing methods of evaluation may be unsatisfactory and ambiguous, for various reasons:

- The fabric pilling rating may vary depending on the standard testing method.
- Assessment and classification is based on the experience of the evaluators and their subjective perceptions.
- Photographic standards representing the pilling scale are inconsistent. For instance, the EMPA reference photos cover the boundary of two classes: 1–2, 2–3, 3–4, and 4–5. This provides the operator with more flexibility for assessment. The ASTM standard, in contrasts, covers a set of 5 images, one for each class.
- A pilled fabric often has distinct pills, ambiguous hairiness, and small pills that are difficult to classify^2^.
- High rubbing force during the test may lead to pills forming that are then torn off. This can result in misevaluations, as the fabric then seems of higher quality^3^.

The subjective nature of pilling intensity classifications has led researchers to seek new more accurate methods, mainly using 2D imaging. Research into automatic pilling assessment techniques began in the late 1980s^4^. Computer analysis focused on counting visible pills and calculating their total and mean areas on a fabric surface, especially based on thresholding^5–9^. Despite the popularity of pilling evaluation methods based on the binarisation threshold, they are often considered unsuitable for fabrics with a predominance of less pronounced pilling features^10,11^. Other researchers developed solutions based on the periodic structure of fabrics and analysis of image spectra created using the Fast Fourier Transform (FFT)^12–14^ or fast wavelet transform (FWT)^15,16^. Mostly, these studies analyzed photographic standards for pilling resistance, while the evaluation of real samples was strongly limited.

Attempts have also been made to reconstruct the three-dimensional surface of fabrics, to detect the symptoms of pilling by segmentation and then classify pilling levels. For this purpose, stereovision solutions such as camera systems were proposed^17,18^. Mendes et al. used a 3D optical system to analyze the degree of pilling. The system consisted of two cameras capturing images of the surface of a fabric sliding under a beam of white light in a dark chamber^18^. Ouyang et al.^17^ used 3D surface images of fabrics obtained using a stereovision system to evaluate the pilling process by detecting individual pills. They are located at local maxima of a fabric surface and then identified by region growing method. Technikova et al.^19^ proposed a gradient field method for obtaining 3D reconstructions based on four initial images (the textile is laterally illuminated from four different sides). The depth map of the fabric surface was restored using the method described by Agraval et al.^20^. Pill-related characteristics were determined from reconstructed 3D peaks representing the pills. These characteristics were used to build a multivariate regression model for predicting the objective pilling grade. Quite recently, Wu and co-authors^21,22^ proposed an objective rating method for pilling based on deep learning with a convolutional neural network (CNN). The CNN method provides an “end-to-end” fabric pilling rating, with automatic learning of features and classification without any manual pattern.

Traditional visualization techniques based on cameras are still more commonly used to test the surfaces of fabrics. Furthermore, Semnani and Ghayoor^23^ used an optical scanner to create a 3D model enabling the measurement of pilling criteria such as the surface area, height, and volume of pills. Yao et al.^24^ used laser range sensing to evaluate pilling by measuring the fabric surface geometry.

In this paper, we propose the use of optical coherence tomography (OCT)^25^ to explore structural changes in textile surfaces associated with pilling behavior. We employ image analysis tools to extract textural features from the OCT images for subsequent multi-group pilling classification. Our method is based on the following assumptions:

1. OCT images of the textile surface are captured with infrared light at a resolution capable of registering fine pilling elements, including outlier fibers and their tiny clumps.
2. Analysis is performed on image texture reflecting fiber density in the pilling layer.
3. The analysis includes the initial pilling phase, which seems adequate due to self-cleaning of pills that sometimes occurs after standard abrasive tests.
4. The template images are only used by the experts for the training phase.

The use of OCT technology is a novelty introduced by the authors, which has not been used previously to assess pilling or fuzz phenomena. This technology, which is typically used for in vivo studies^26^, is practically not applied in textile research. Exceptions include studies of fabric weave patterns^27,28^ and the authors’ own pilot studies on the effectiveness of fabric surface laser modification^3,29^.

Known methods of 3D fabric imaging by stereovision^17,18^ provide lateral and vertical resolutions about 200$\div$300 $\upmu$m and 20 $\upmu$m, respectively. Therefore, their fabric classifications are based on counting the pills, formed during traditional pilling tests, which are large enough to be detected inside images. The proposed method uses resolutions at least twenty times higher, what enables to detect and assess thinner bundles of fuzz fibers. Additionally, the infrared radiation used in the new method penetrates the pilling layer that is semi-translucent to it, what allows the detection of many fibers partially hidden to the visible light. Hence, our approach is significantly different from previous solutions, because it is based on the computer classification of the textural features of the 3D pilling layer instead of counting the pills. It can detect both pills existence and fuzz appearing at the early pilling stage. As previously demonstrated^3^, there are fabrics, where the correct number of pills cannot be obtained and you must use the early pilling phase to evaluate the pilling grade properly.

## Methodology

### Pilling resistance assessment framework

**Figure 1:**
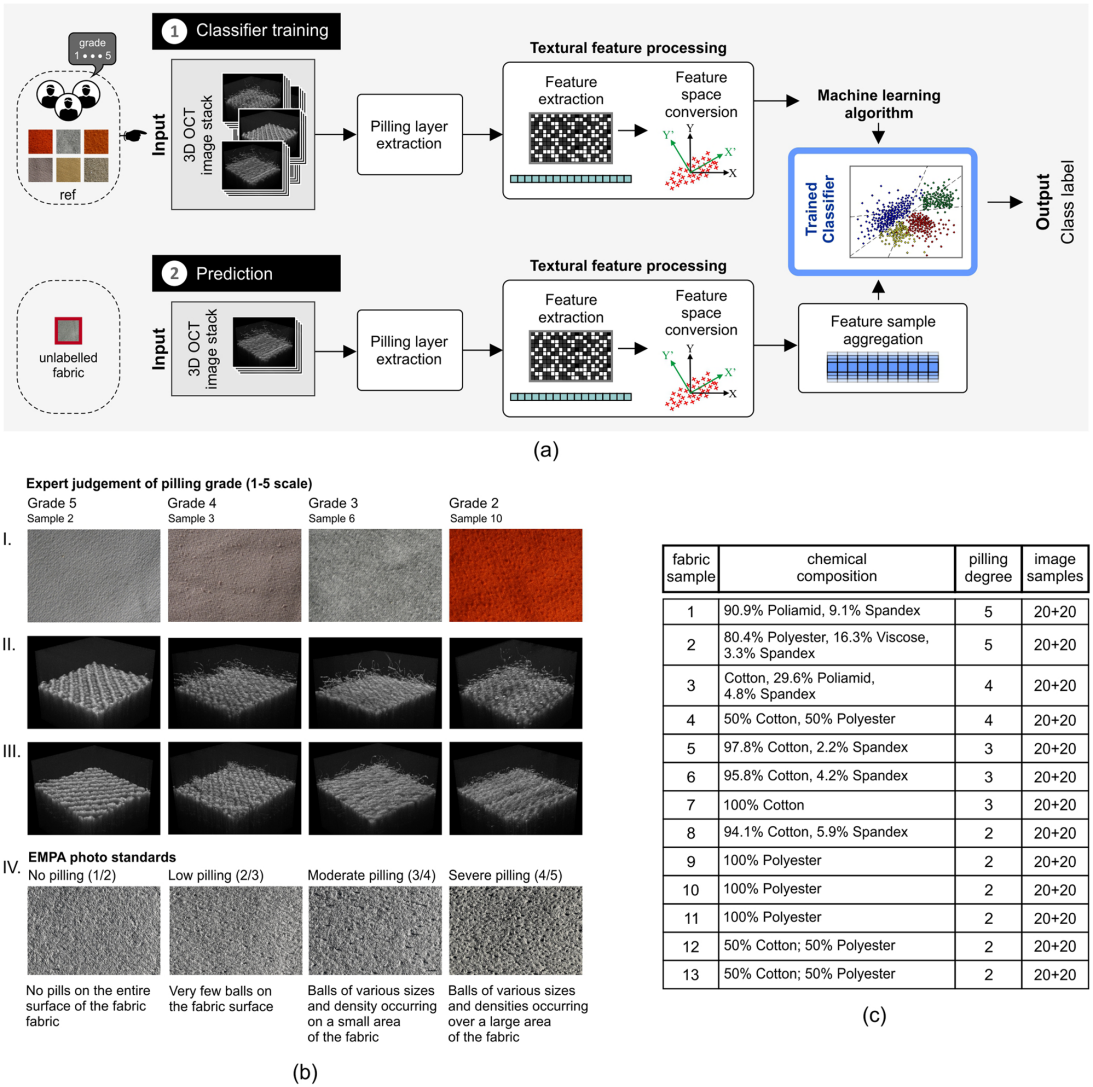
Illustration of the proposed method for assessing pilling resistance. Textural features associated with pilling behavior are extracted from OCT volumetric scans. Pilling tendency is evaluated using PLS-DA, LDA, and SVM classification methods. To predict the degree of pilling, a virtual sample of each fabric is introduced, with the features averaged from images taken of a single fabric (**a**). The entire test set of scans consists of fabrics with pilling symptoms of various degrees of severity (**b**) and chemical compositions (**c**). Pilling grades are given by experts on the basis of the EMPA photographic standard for textiles, No. SN 198525. The images in rows II and III from subset (**b**) illustrate the surfaces of exemplary fabrics with different pilling classes, visible in OCT images after custom and Martindale abrasion tests, respectively.

Figure 1a presents the proposed system of pilling degree classifier of fabrics. This system can work in training and prediction modes visualized as two paths 1, 2 of data processing in the block diagram. The *Input* module in each path includes an OCT scanning system, which produces 3D fabric images with the pilling layers (see “Description of the experimental environment” section). In the training mode several hundred images are created from all fabric samples shown in Fig. 1c, while in the prediction mode one or five images of a single fabric are required. The fabrics are classified after two different abrasion tests:

- custom gentle abrasion test to obtain initial pilling in the form of individual fuzz fibers, as shown in Fig. 1b, row II. This custom test will be referred to as T1.
- Martindale apparatus test, referred as T2, to obtain pills, which allowed experts to label pilling grade of the fabrics on a five-point scale corresponding to increasing resistance to pilling.

After the machine test T2, in the case of fabrics with a tendency to pilling, OCT images may contain fewer individual fibers than after the manual test, but usually contain additional structures of fibers clumped together (Fig. 1b, row III). The proposed system of assessing pilling resistance aims to capture these differences in terms of the textural features. In both paths shown in Fig. 1a the pilling layer is extracted from the OCT volumetric images using the *Pilling layer extraction* module, detailed in “Extraction of the pilling layer from an OCT fabric image” section. It enables selection of the pilling region only, without the fabric layer below it. *Textural feature processing* block performs the calculations of pilling image textural characteristics such as Haralick^30^ or fractal features^31^ and image lacunarity properties^32^. This is described in detail in “Extraction of textural features” section. After the extraction of features they are cleaned of outliers, scaled (“Extraction of textural features” section) and before the classification reduced to the limited number of mutually independent components as the *Feature space conversion* task (more in “Feature space and sample correction” section). *Trained classifier* block in the training mode performs machine learning algorithm to determine proper values of the internal parameters. The tested algorithms are SVM^33^, LDA^34^ and PLS-DA^35,36^. In the prediction mode, the classifier anticipates the pilling grade of the fabric from the features of single image sample or the aggregation of 5 samples. “Feature sample aggregation”, explained in section, is added to improve classification accuracy. It averages textural features of 5 image samples from the same fabric before prediction.

### Abrasion tests

The purpose of the custom test T1 is to cause fiber protrusion, in the form of fuzz, over the fabric surface. During the test, each type of material was manually rubbed using a brush with hard fibers for 15 s whilst maintaining a constant pressure. The pressure force of the friction surface on the material was set at $2\,\mathrm {N}$, controlled by a strain gauge. Test T2 was carried out using a Martindale device and the principles of forced pilling described in PN-EN ISO 12945-2: 2021-04 and PN-EN 12947-1:2002. A felt disc with the tested material was mounted on an instrument table. The Martindale device head was moved along tracks at a constant speed in a Lissajous curve relative to the stationary table, under a load of $4\,\mathrm {N}$. After 5000 head movements, the samples were evaluated by three experts according to ASTM D 3511-08. The final assessed pilling grades were the arithmetic averages of the partial grades given by the evaluators, rounded to the integer scale of 1 to 5.

After each abrasive test, 20 spatial image samples were acquired for each of 13 fabric types shown in Fig. 1c. A total of 260 OCT images for each abrasion test were used to build and test the various pilling models.

### Description of the experimental environment

**Figure 2:**
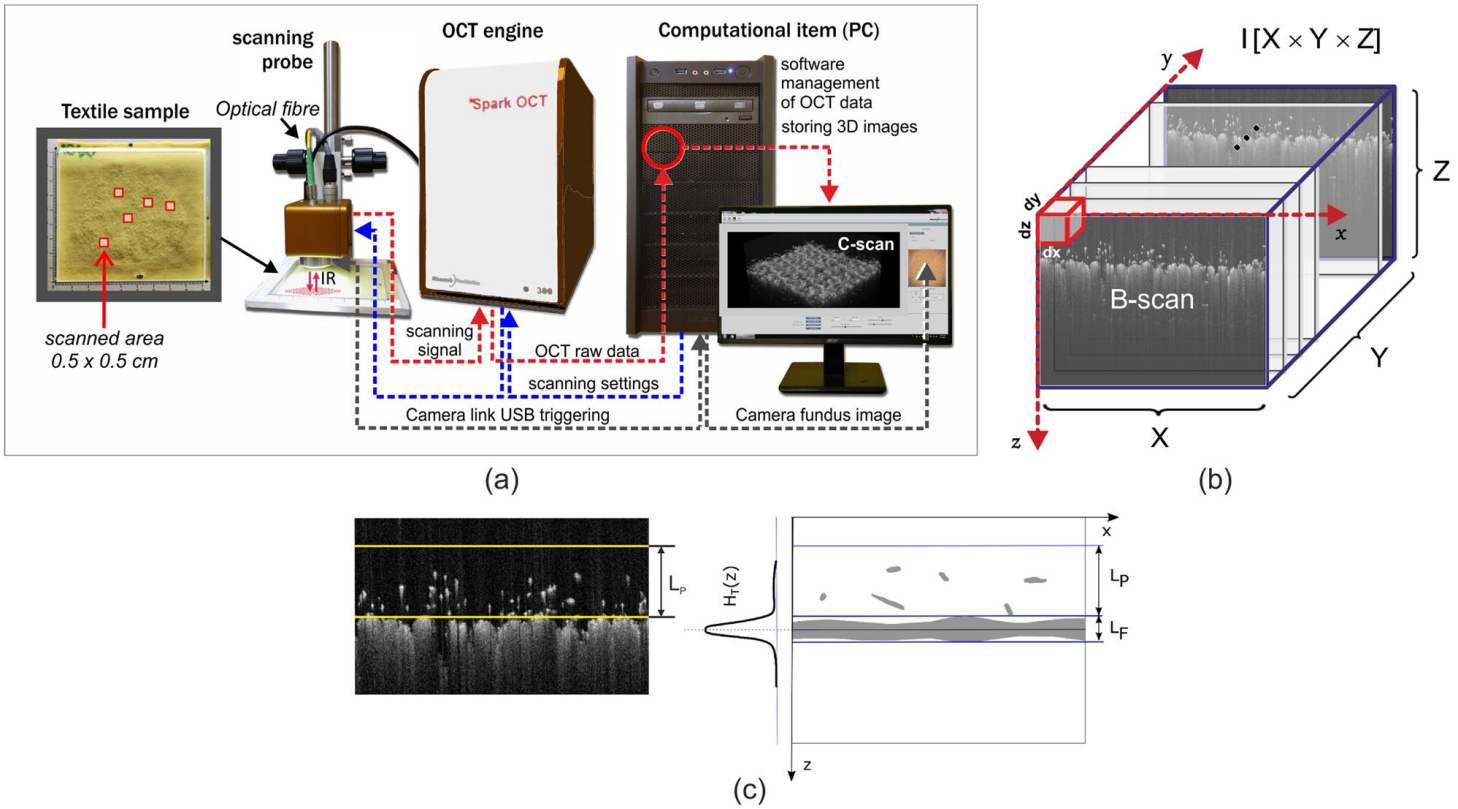
Acquiring information of the fabric surface. (**a**) HR-Spark OCT 1300 nm OCT setup. The engine module contains a Fourier domain (FD) Michelson’s interferometer, where interference is obtained using a short coherence light scanning source. The sample arm of the interferometer is equipped with an infrared scanning head in the OCT Scanning Probe. (**b**) Stack of acquired B-scans equivalent to OCT volumetric data. (**c**) Illustration of the layer model, applied to an OCT image of a pilling fabric. A single B-scan with fabric and pilling layers is visible. $L_F$ – fabric layer. $L_P$ – pilling layer. $H_T(z)$ – Hough transform in *z* direction.

Images of the fabric samples after abrasion tests were acquired using the Spark OCT 1300 tomographic laboratory system (Wasatch Photonics Inc., Morrisville, NC, USA) (Fig. 2a). The scanning head of this system emits a laser beam of infrared light, which penetrates the fabric with its pilling layer. The registered 3D images are created by infrared signal reflected from the fuzz fibers, pills and the fabric surface, which are semi-transparent for the infrared radiation. The intensity of the signal reflected at each location (*x*, *y*, *z*) is picked up by the head receiver and stored in the form of a three-dimensional image array (Fig. 2b) written in a DICOM-type file. Every OCT volumetric image is acquired in a raster of $512 \times 512 \times 640$ voxels in the Cartesian coordinate system. Each voxel has dimensions equal to $dx=10.2\,\upmu \mathrm {m}$, $dy= 9.6\,\upmu \mathrm {m}$, and $dz =5.4\,\upmu \mathrm {m}$. The image covers a volume of $0.5 \times 0.5 \times 0.4 \,\mathrm {cm}$, containing both the fabric layer and the space around it. The scanning process collects the image from two-dimensional B-scan frames, acquired in real time. To facilitate detection of the fabric layer inside the OCT image, the fabric surface is kept in a horizontal orientation inside each B-scan by adjusting the tilt of the scanning head. The algorithms for extracting the pilling layer and computing the textural features were written in Python language. The proposed classifier models and validation algorithms were prepared in the R language using the *mixOmics* package^37^.

### Extraction of the pilling layer from an OCT fabric image

Pilling assessment was based on a three-dimensional model of the pilling layer above the fabric surface, which was extracted from the acquired OCT image *I*(*x*, *y*, *z*).

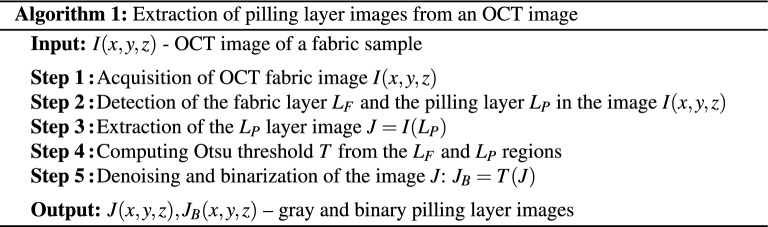



Algorithm 1 represents the extraction method. Applying the Hough transform^38^ to the image *I* previously binarized by Otsu thresholding^39^ allows detection of the borders of the fabric layer $L_F$ and the pilling layer $L_P$, as illustrated in Fig. 2c. The layer detection method was developed by the authors in a previous study, and was originally described in an article by Sekulska-Nalewajko et al.^3^. The image *J* including only the layer $L_P$ is extracted from the acquired image *I*. It is then denoised by median filtering^40^ and binarized into $J_B$ before its textural properties are computed. The Otsu binarization threshold is evaluated based on the fabric layer $L_F$ and part of the pilling layer $L_P$ of equal height, to provide similar amounts of light and dark voxels before thresholding.

### Extraction of textural features

The pilling layer consists of protruding fabric fuzz and pills generated during the fabric friction tests. In the spatial image *J*, the voxels with higher and lower gray levels correspond to more or less light scattered by the fabric material migrating into the pilling layer. Statistical properties derived from the Gray Level Co-occurrence Matrix (GLCM) were related to the intensity of the voxels and thus indirectly to the presence of pilling fibers. The Haralick textural features $H_1,\ldots ,H_{13}$ of the image *J* were used to assess pilling^30^. We also used the features of the fractal dimension, border mean, and border area proposed for Segmentation-based Fractal Texture Analysis (SFTA)^31^. Moreover, we calculated the fraction of fiber pixels, detected in the binary image $J_B$ obtained by global thresholding of the image *J*. The pilling voxel fraction $f_P$ is defined in Eq. (1) as

1 $$\begin{aligned} f_P=\dfrac{1}{V}\sum \limits _{x,y,z}J_B(x,y,z), \end{aligned}$$

where *V* is the volume of the image $J_B$ expressed in voxels. The fractal dimension *D* proposed here is based on the box-counting (Minkowski) dimension $dim_{box}(S)$ applied in 3D space according to the formula in Eq. (2)

2 $$\begin{aligned} dim_{box}(S)=\lim _{\epsilon \rightarrow 0}\dfrac{log N(\epsilon )}{log(1/\epsilon )}, \end{aligned}$$

where $\epsilon$ denotes the side length of the sliding cube, *N* is the number of cubes, and *S* is a 3D data set of the image voxels. The dimension *D* is estimated by the least squares method using the linear equation $y = Dx + A$, where $y = log(N)$ and $x = log (1/\epsilon )$^41^. To obtain the value of *D*, gliding boxes of size $[\epsilon _i \times \epsilon _i \times \epsilon _i]$ are used. The number of boxes $N_i=N(\epsilon _i)$ containing at least one bright voxel in the border image $\Delta (J_B)$ is computed for each box of size $\epsilon _i = s, s/2, s/4, \ldots , 2$, starting with an initial size *s* equal to the smallest image dimension rounded to the power of 2. The data $(x_i, y_i)$ are computed for each box size $\epsilon _i$ according to the Eq. (3)

3 $$\begin{aligned} y_i=log(N(\epsilon _i)),\quad x_i=log(\epsilon _i). \end{aligned}$$

The least squares method of regression^42^ is then used to compute the linear approximation of the data. The fractal dimension given in Eq. (4)

4 $$\begin{aligned} f_D=-D \end{aligned}$$

represents the negative slope of the line *y*(*x*). Other textural features sensitive to the amount of pilling are the border mean value $b_m$ and the border area $b_{ar}$. The $b_m$ can be computed as the average brightness of the layer image $J=I(L_P)$ on the borders of protruding fibers in $J_B$, as in Eq. (5)

5 $$\begin{aligned} b_m=\dfrac{1}{N_{\Delta }}\sum \limits _{i=1}^{N_{\Delta }} J(\Delta ), \end{aligned}$$

where $\Delta (J_B)$ denotes the border image obtained from $J_B$ and $N_{\Delta }$ is the number of border voxels. The border area $b_{ar}=N_{\Delta }$ includes all $J_B$ border voxels.

Lacunarity analysis^32^ of the pilling layer image $J_B$ allows the dispersion of textural content to be assessed at different scales. The lacunarity $\Lambda _b$ with the scale *b* is defined by Eq. (6)

6 $$\begin{aligned} \Lambda _b(J_B)=\dfrac{\sigma ^2(J_B)}{\mu ^2(J_B)} +1, \end{aligned}$$

where $\sigma$ (standard deviation) and $\mu$ (mean) refer to changes in the sum of $J_B$ white pixels in a sliding window. Lacunarity $\Lambda _b$ is measured in the image $J_B$ with a cube moving window of side *b* less than the image smallest dimension, which is the height.

To address the issue of possible data outliers based on 1.5 times the Inter Quartile Range (IQR), outlier observations were capped by replacing them with the values of the 5-th or 95-th percentiles, respectively, for outliers exceeding the lower or upper limits of the decision range. As the discussed textural features can be expressed on different scales, all of them should be standardized to balance their impact on the pilling grade. The input scaling process consists of centering and normalizing each feature independently, according to the formula in Eq. (7)

7 $$\begin{aligned} x_i^*=\dfrac{x_i-\bar{x}}{\sigma _x}, \end{aligned}$$

where $\bar{x}$ and $\sigma _x$ denote the mean value and standard deviation of the feature *x* in the set of all fabric images *I*, and where $x_i^*$ is the normalized value of the *i*-th feature sample. Each feature with $\sigma _x=0$ is excluded from the discrimination set, as it cannot distinguish fabric samples by their pilling degrees.

### Feature space and sample correction

**Figure 3:**
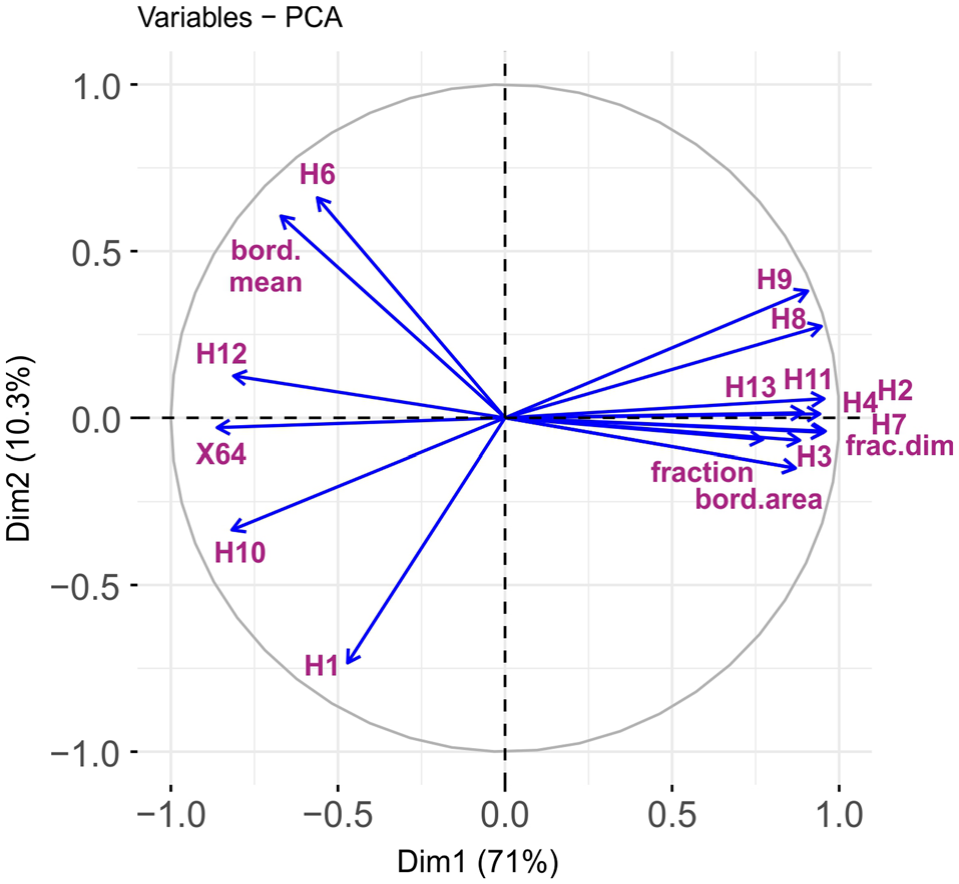
Input textural features and their contributions to the principal axes.

The input data for the machine training algorithm is the matrix $X[n \times p]$ of *n* training sample rows with *p* textural features, or the matrix $Z[m \times p]$ of *m* predicted sample rows. For the proposed classification algorithms, except PLS-DA, the sample features determined in columns of *X* or *Z* are converted into the principal component columns of $X_c$ or $Z_c$, respectively, using the principal component analysis (PCA) transform shown in Eq. (8).

8 $$\begin{aligned} X_c=X \times W,\quad Z_c=Z \times W, \end{aligned}$$

where $W[p \times p]$ is the matrix of principal component vectors computed from the covariance matrix $X^TX$. These vectors correspond to the PCA loadings shown in Fig. 3 on the plane of two major components (*Dim*1, *Dim*2). The many loading vectors of similar directions observed in this diagram confirm the fact that some training features in *X* can be correlated, and therefore their conversion to the PCA space of orthogonal components is fully justified. The reduced matrices $X_c[n \times q]$ or $Z_c[m \times q]$ preserve only the first *q* component columns of the originals $X_c$ or $Z_c$. The columns are ordered by decreasing eigenvalues, which allows most of the matrix information to be retained. Using PLS-DA classifier excludes the PCA transform. The matrices $X_c[n\times q]$, $Z_c[m\times q]$ containing the training data and predicted data components are computed internally by the PLS-DA classifier.

### Feature sample aggregation

In the aggregation stage, *m* samples in the array $Z_c[m \times q]$ (or $Z[m \times p]$ for the PLS-DA classifier) are averaged along the sample rows as in Eq. (9):

9 $$\begin{aligned} \overline{z}_{c(j)}=\dfrac{1}{m}\sum \limits _{i=1}^{m} z_{c(i,j)}, \;\; j=1,\ldots ,q \quad \mathrm {or} \quad \overline{z}_{(j)}=\dfrac{1}{m}\sum \limits _{i=1}^{m} z_{(i,j)}, \;\; j=1,\ldots ,p \end{aligned}$$

The component row $\overline{Z}_c[1 \times q]$ (or feature row $\overline{Z}[1 \times p]$ for the PLS-DA) is then applied to the input of a previously trained classifier, to predict the degree of pilling. This operation is labeled as the “Feature sample aggregation” module in Fig. 1a.

### Pilling degree classifiers

We assessed the ability of three popular classification methods to distinguish degrees of pilling. The methods were as follows:

- Linear Discriminant Analysis (LDA)^34^,
- Partial Least Squares Discriminant Analysis (PLS-DA)^35,36^,
- Support Vector Machine (SVM)^33^.

The LDA technique was selected because it has a closed-form solution that can be easily computed, works well in practice, applies to multiple classes, and has no hyperparameters to tune. To apply LDA all classes corresponding to the degree of pilling were assumed to be linearly separable by hyperplanes in the component space. Gaussian data distributions, and the same covariance matrix shared among all classes are required for correct classification. The PLS-DA algorithm was used for predictive modeling of the degree of pilling, as well as discriminative selection of input components avoiding PCA transformation on input. We restrict our attention primarily to predictive modeling. Theoretically, PLS-DA combines dimensionality reduction and discriminant analysis in one algorithm, and is especially suitable for modeling high-dimensional data. Moreover, PLS-DA does not assume that the data fit a particular distribution, and is therefore more flexible than other discriminant algorithms, including LDA. We decided to use SVM as a non-linear classifier, to compare its results with the results for LDA and PLS-DA. SVM searches for the hyperplane with the largest minimum margin between training samples of a given pilling class and other classes. It also searches for a similar hyperplane that correctly separates as many instances as possible. The cost parameter *C* determines how much more important is the latter type of hyperplane. New, unlabeled samples are then mapped onto the same space and predicted to belong to a class based on the side of the margin on which they fall. Non-linear classification is possible using the so-called kernel trick, whereby input data are implicitly mapped onto high-dimensional component spaces. The radial basis function (RBF) was selected as a kernel defined by Eq. (10):

10 $$\begin{aligned} K(u,u')=exp(-\gamma \Vert u-u'\Vert ), \end{aligned}$$

where *u* and $u'$ represent row vectors of the component matrix $X_c$ or $Z_c$, $\gamma =1/(2\sigma ^2)$ and $\sigma ^2$ is the kernel function variance.

## Experimental and analysis

### Pilling classification results

The discussed method of pilling prediction was tested on a training set of textural feature samples, collected in the form of a matrix $X[n \times p]$ associated with the vector *Y*[*n*] of established pilling degrees, for $n=260$ samples and $p=17$ features. Only the classes from 2 to 5 on the five-point scale of pilling degrees were analyzed, because none of the fabrics showed evidence of the most severe (grade 1) pilling (Fig. 1c). The suitability of the classifiers to determine the degree of pilling resistance was verified by the classification error, as defined in Eq. (11) using confusion matrix $M_{CF}$^43^ .

11 $$\begin{aligned} err\_rate=\dfrac{\sum \limits _{i=1}^{N_c}\sum \limits _{\begin{array}{c} j=1 \\ j\ne i \end{array} }^{N_c} M_{CF}(i,j)}{\sum \limits _{i=1}^{N_c} \sum \limits _{j=1}^{N_c} M_{CF}(i,j)}=1-AC, \end{aligned}$$

where *AC* denotes the classification accuracy. Two methods of cross validation were applied to evaluate the classification error rate:

- Leave one out (LOO)^44^ predicts a single sample randomly selected from the input matrix *U* of feature components, which is excluded from the training set.
- Leave mean out (LMO) predicts a sample mean computed from five randomly selected sample rows of the input matrix *U*, which are excluded from the training set.

Both LOO and LMO are hold-out stratified cross validation methods. In the case of LOO, one sample is randomly selected from each type of the four considered pilling classes (from 2 to 5). In the case of LMO, five samples are selected randomly in the same way. Stratification of the cross-validation solves the problem of our imbalanced dataset, with samples per fabric pilling class changing from 40 to 120. During validation, the sample random selection for each pilling class is repeated 500 times. Validations were performed for fabrics after our custom pilling test T1 and the Martindale test T2.

**Figure 4:**
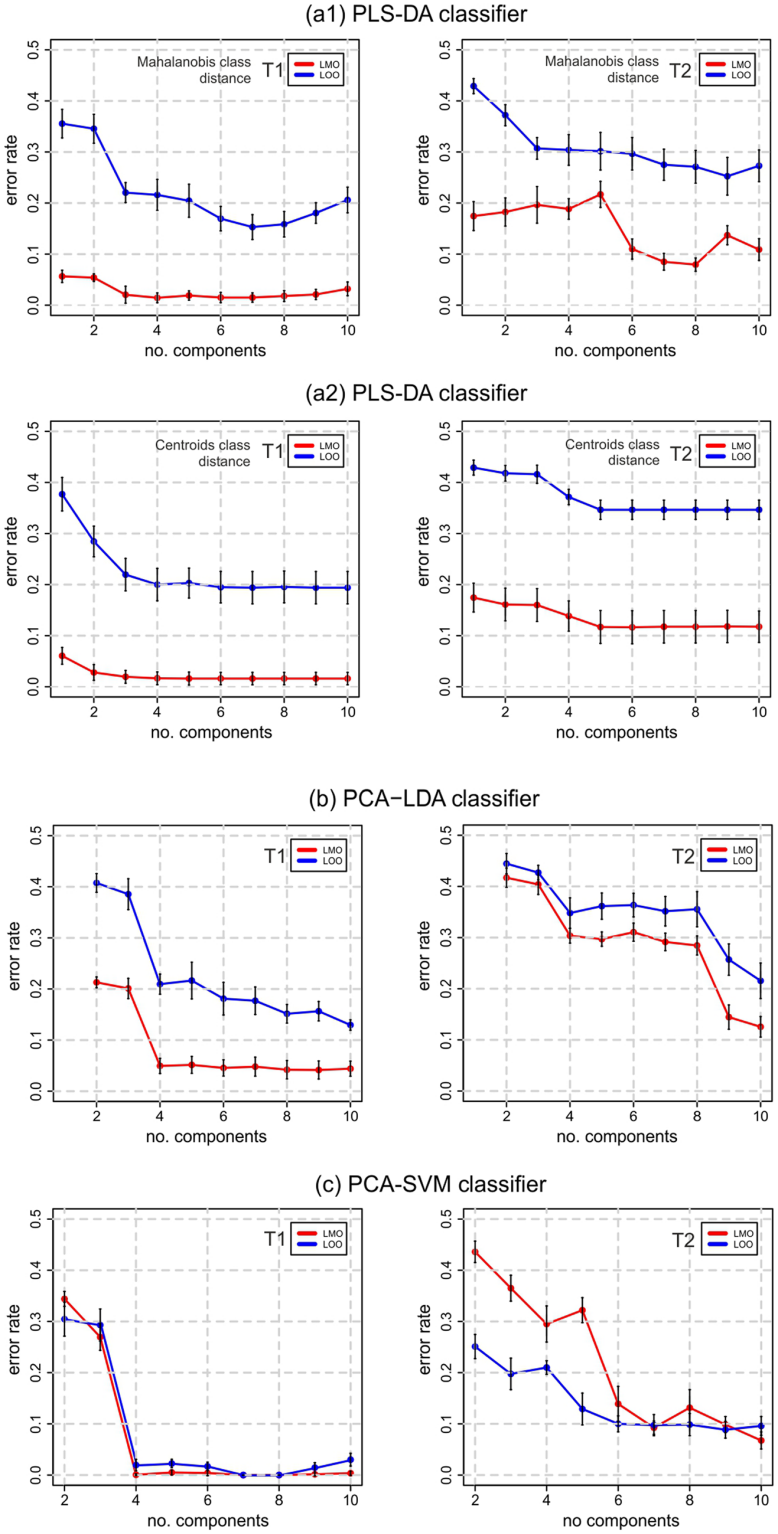
Mean error rates for pilling grade prediction. LOO—Leave-one-out validation, LMO—Leave-mean-out validation. In the case of the PLS-DA classifier, two methods of measuring the distance to the centroids of the pilling classes were tested: (**a1**) Mahalanobis distance and (**a2**) centroid distance.

The computed rates of prediction error for each of the considered classifiers are presented in Fig. 4 as functions of the textural component number. Each data value presented in a plot is the mean of prediction error rates computed from 10 series of 50 validations. The error bars of plot nodes illustrate the error rate standard deviations within the series. To prevent overfitting, no more than 10 input components are considered. This provides at least four times the number of samples than the number of components in a pilling class. In the plots, the error rates generally tend to decrease with the number of components. This is because more data dimensions were used to train the classifier. The classification error rates are much larger for T2 than for T1 test. In the case of the LDA classifier, after T1 and LMO validation the $err\_rate$ stabilizes at about $5\%$ starting from four input PCA components. For LOO validation, the error rates do not fall below $12\%$, even when 10 components are included. The classification errors after T2 are even more than twice as high compared to those after T1, especially when over 4 components are used. The accuracy obtained for the PLS-DA classifier, shown in Fig. 4a, depends not only on the validation method but also on the distance measure between pilling classes. The lowest errors for PLS-DA of $2\%$ were observed after T1, starting from four components for the LMO centroid distance.

The highest accuracy, of more than $99.9\%$ and $98\%$ for LMO and LOO respectively, were achieved for the nonlinear SVM classifier using the radial basis kernel function. This required tuning the RBF parameters $\gamma$ and *C*, depending on the number of input feature components. The sets of these parameters maximizing LOO validation accuracy for $2\div 10$ input feature components are listed in supplemental Table S1. For example, selection of the first five components and LMO validation after T1 provides the lowest error rate for RBF $\gamma =0.5$ and the cost parameter $C=20$. The computed SVM error rates are illustrated in Fig. 4c. Starting from four input PCA components, $err\_rate$ falls to below $0.5\%$ for LMO prediction after custom pilling test T1. After the Martindale test T2, pilling degree classification is much more difficult for the SVM classifier, and the error rate does not drop below $8\div 10\%$ even with 10 components.

**Figure 5:**
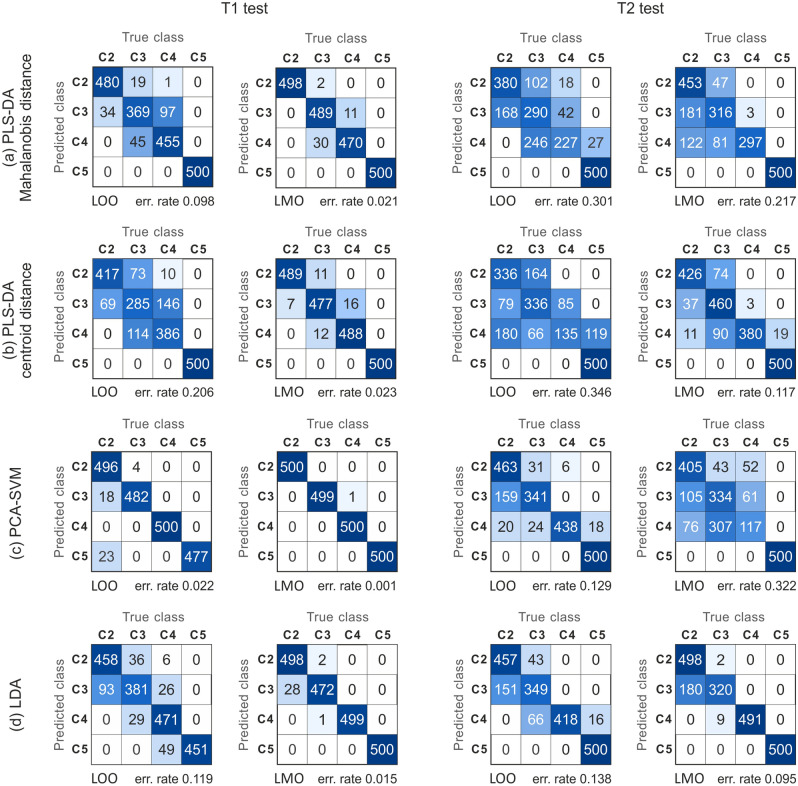
Confusion matrix for the tested classifiers with two textile data sets (the same textiles after custom abrasion test T1 and after the standardized Martindale abrasion test T2) and different validation methods (LOO, LMO). For the PLS-DA classifier, the matrices were computed based on the Mahalanobis distance as well as the centroid distance of the first five output components. The SVM classifier confusion matrices were computed after dimension reduction for five PCA components.

Example confusion matrices for the first five PLS-DA explanatory variables or the first five PCA components sent to the SVM classifier are included in Fig. 5. The set of training samples provided for each pilling class is at least several times larger than this number of components, to avoid overfitting at the training stage. In the case of LDA classifier, the reduction of features to 5 PCA components significantly increases error rate of the prediction. Therefore, the confusion matrices for this classifier are presented without PCA. The matrix rows correspond to the predicted pilling classes and their columns correspond to the actual classes determined by experts. The matrices thus illustrate the distribution of prediction outputs and simultaneously the prediction errors for different pilling classes. All confusion matrices for the fabrics after T1 with LMO or LOO validation are diagonally dominant, which means that most of the predictions are correct. LMO validation of fabrics subjected to T1 guarantees that the prediction error does not exceed the neighboring classes on the pilling resistance scale. The number and spread of false predictions are much greater after T2 than after T1.

**Figure 6:**
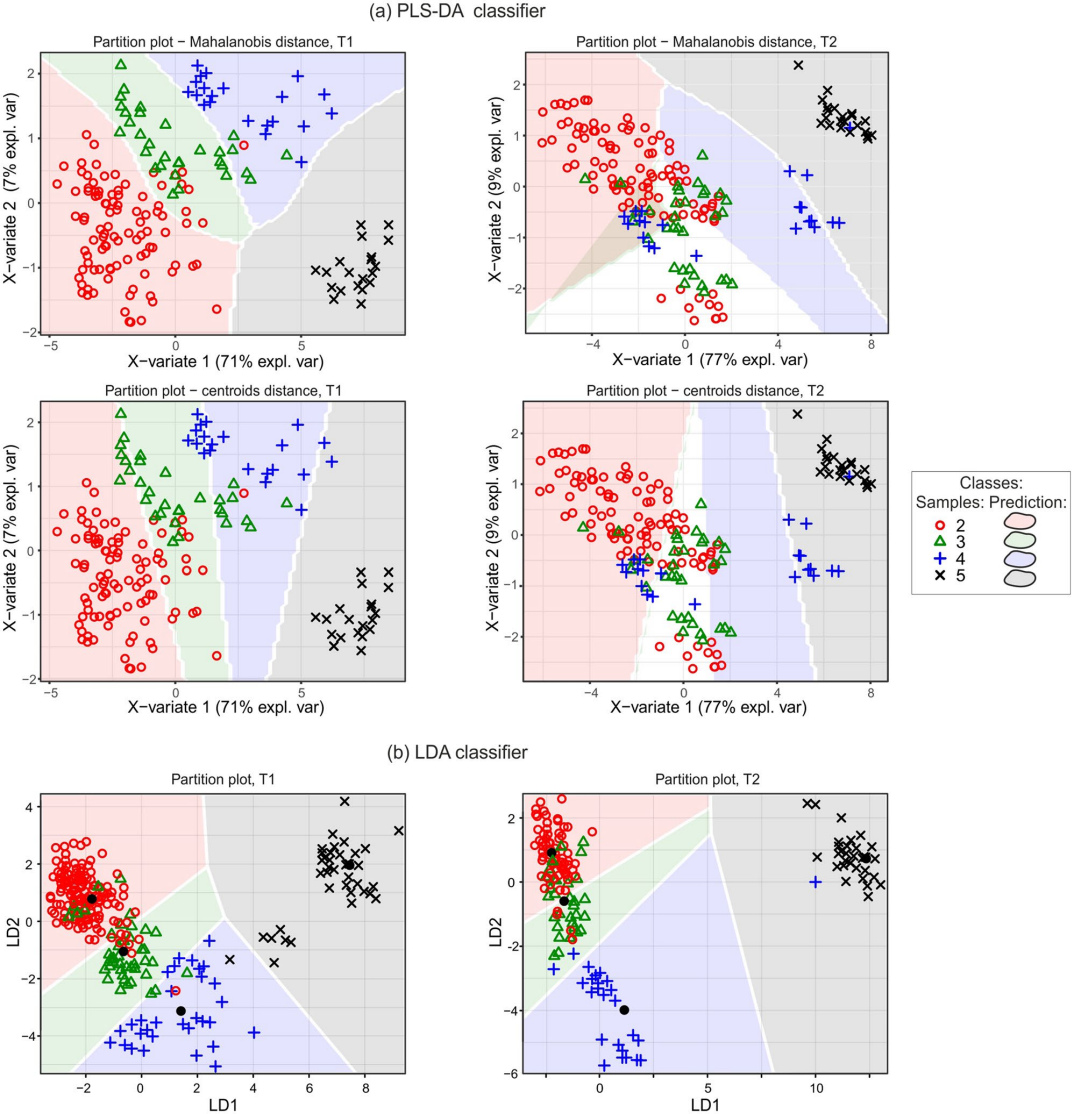
PLS-DA and LDA classifier prediction results for textural feature samples in the plain of the first two linear discriminant dimensions.

Figure 6 illustrates the distribution of fabric feature samples on the plain of the first two explanatory variables, exposed on the background of their predictions. The feature data obtained after T1 fit much better to their predicted regions than the feature data for T2, for which white regions of no prediction or grayed regions with ambiguous predictions can be observed.

**Figure 7:**
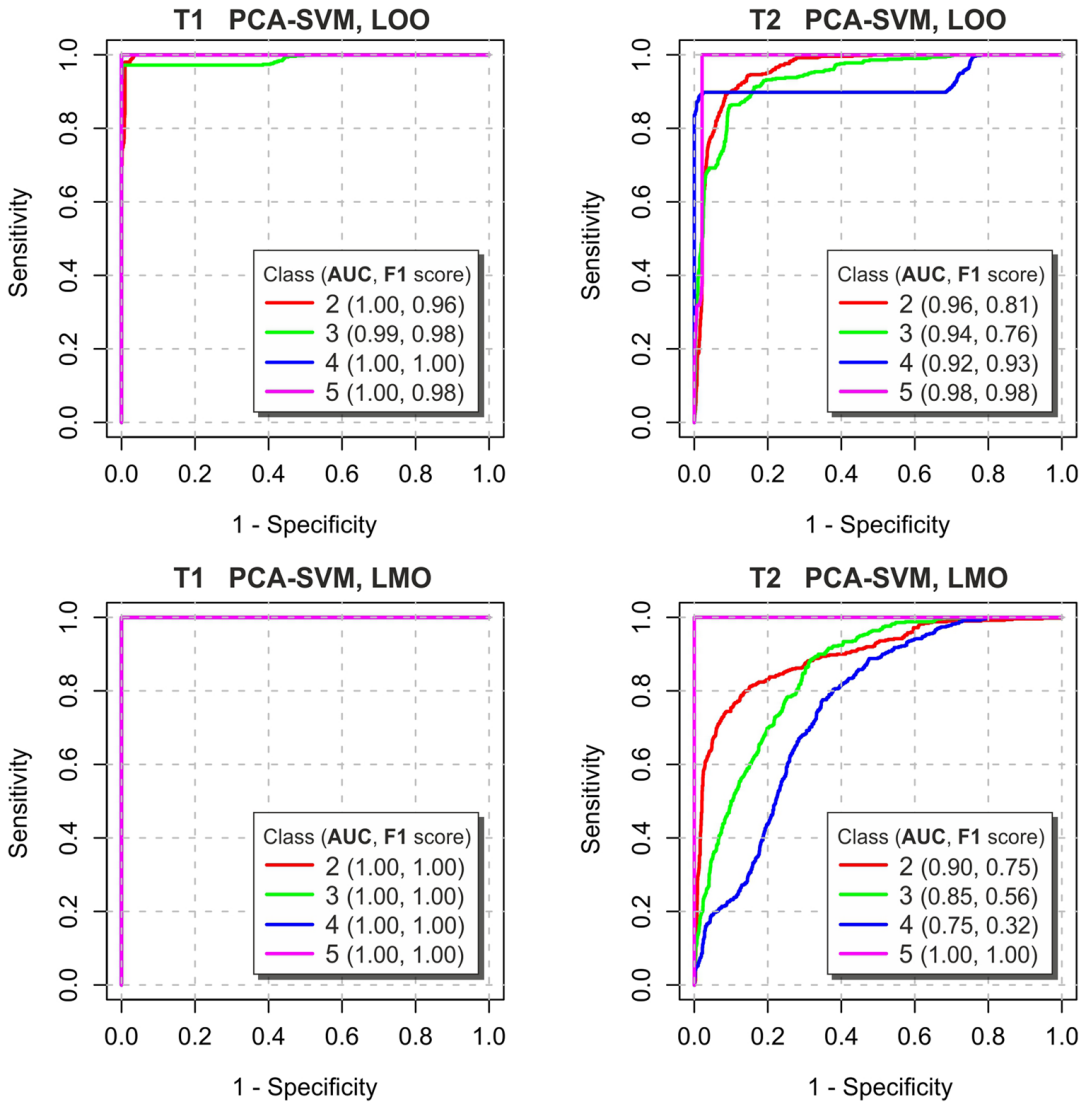
ROC curves type OvR for each pilling grade class obtained from the nonlinear SVM classifier of the 5 PCA components, after the abrasion tests T1 and T2. Optimal cut-points on the curves are given as Sensitivity and Specificity in supplemental Table S6. *AUC* and *F*1 values are presented in the legend.

Moreover, the Receiver Operating Characteristic (ROC) curves^45^ have been computed using the method One vs. Rest (OvR), which compares each class against all the other. ROC plots in Fig. 7 illustrate *Sensitivity* against $1-Specificity$^43^ changes of each fabric class at different classification thresholds. After the test T1 for SVM classifier the ROC curves for all piling classes go very close to the point (0, 1), as confirms the value of Area Under the Curve ROC (*AUC*) above 0.99. The data to prepare these plots have been taken as raw probability outputs from the classifier. Also *F*1-scores of individual fabric classes have been computed (Fig. 7) to assess the quality of sample identification of each pilling class. The *F*1-scores of all classes after T1 abrasion test are very high and reach maximum values for LMO validations. According to *F*1-score the fabric classes *C*2 and *C*3 are distinguished only marginally worse after abrasion test T1 and LOO validation.

The ROC curves after the Martindale abrasion have lower *AUC* values: 0.95 and 0.87 on average for LOO and LMO validations, respectively. There is also a visible deterioration for *F*1-score for all pilling classes except *C*5, because it represents fabrics most resistant to pilling with no fibers or pills, which stands out well among others. All this proves that the proposed fabric classification works better for custom abrasion test T1 than for the traditional T2.

Supplementary material includes sensitivity, specificity and *F*1 results for all considered classifier, and ROC curves for LDA and PLS-DA classifiers. After the abrasion test T1 the classifier PCA-SVM achieves better accuracy than LDA, PCA-LDA or PLS-DA (Table 1). After the abrasion test T2 the classifier LDA without feature reduction has the best accuracy of 0.904 and average results of *F*1 equal to 0.902 for LMO. The presented results indicate that the PCA reduction of the computed textural features left after the Martindale abrasion significantly deteriorates the classification quality. For PLS-DA ROC and AUC criteria (supplemental Figure S2) obtained with the *mixOmix* :  : *auroc* R function may not be in agreement with the PLS-DA performance, as the LMO and LOO prediction threshold in PLS-DA is based on specified distance. These curves use a cutoff that maximizes specificity and sensitivity rather than this distance.

## Discussion

The key challenge in traditional and objective methods of pilling evaluation is how to extract both hairiness and pilling from the fabric surface pattern. Hairiness often has thickness in the micron range, which is why we propose the use of OCT as an imaging technique. Unlike traditional methods, OCT can provide direct information about the depth of the fabric surface.

In our study, we considered the possibility of automatic pilling evaluation in the early and late stages of pilling, using several classifiers. Early pilling is generally characterized by an increase in the amount of protruding fibers and by slight degradation of the fabric surface. This stage is characterized in the literature by the initial formation of pills^46,47^. The fabrics were graded by experts after machine testing. They generally maintained their human assigned categories when evaluated by the proposed computer classification algorithms. However, higher accuracy was obtained in the case of textiles at the early pilling stage.

For classification of the early stage of pilling, the highest accuracy at over $99\%$ was provided by the non-linear SVM classifier, starting from four input components. Slightly larger prediction errors of about $2\%$ occurred in the case of PLS-DA with a centroid or Mahalanobis distance. The results for PLS-DA at the maximum distance were not considered, because it gave much worse predictions than for the other two distances. The considered results were obtained for the proposed aggregation of features of five samples randomly drawn from the same material. Predictions of single fabric samples showed errors of between $10\%$ and $20\%$ for linear classification and much lower errors of about $2\%$ for the non-linear SVM classifier. However, the erroneous predictions indicate distant classes in the pilling scale, which makes the results unreliable (Fig. 5c, test T1).

The high accuracy of the SVM classifier with its $\gamma$ and *C* parameters fine-tuned indicates that the pilling degrees are not fully linearly separable. According to the literature, SVM classifiers can provide satisfactory results for pilling assessment on the basis of hairball characteristics. Yang et al.^48^ used the SVM classifier with the RBF core function to identify the pilling grade. The tested parameters were captured automatically using deep principal component analysis (DPCA) from flat images of fabrics treated with a traditional Martindale device. The accuracy of SVM prediction was $99.7\%$.

In our study, OCT fabric pilling images after the Martindale machine test were classified with large errors of between $10\%$ and $30\%$. The predictions are scattered over many true classes, and there is no guarantee that most of the predictions in the validation tests fall into the correct class. This is because the fibers of the fuzz are not evenly dispersed in the layer above the fabric, as they are after the early-pilling test. Instead, they are rolled up into pills adjacent to the fabric surface. Quantitative changes in such forms are much more difficult to detect by means of infrared laser scanning. On the other hand, it is easier for an expert to count the number of pills and assess the fiber fuzz residues on the surface of a fabric properly illuminated by visible light.

We also evaluated the performance of the classifiers using the reliability index $\eta$ proposed by Furferi and co-workers^49^. This index takes into account the correction of “false” indications, by assigning separate definitions (different weights) for overstated and underestimated scored compared to expert judgments. A misclassification to a worse class is conservative, and does not have such significant commercial ramifications as false-good results. On the other hand, a serious erroneous classification overstating the quality of the fabric leads to a significant reduction in the reliability index. The index equation and the weights of its components are given in^49^. Changes in the reliability index for the classifiers and validation methods used in our study are shown in Table 1. The index values vary from $63.2\%$ to $91.4\%$ for samples after abrasion with the Martindale machine. In comparison, Furferi and co-workers reported a reliability index equal to $88.52\%$ in a study on pilling classification performed with their artificial neural network (ANN). The study used a feedforward backpropagation ANN trained on 11 statistical and brightness-related parameters, characterizing a 2D fabric image after the Martindale test. Their two classification procedures, based on a self-organizing feature map (SOFM) and k-means clustering, gave worse reliability index values of $43.53\%$ and $65.19\%$, respectively. Based on the reliability index, it can be concluded that the classifiers performed well on the samples after both abrasion tests, although there were a large number of incorrect classifications in the case of the sample set after machine testing. This was mainly due to the fact that most of the discrepancies between the classifier indications and the expert judgments were assignments of samples to a lower class (worse pilling). The reliability index values obtained for the proposed early pilling test T1 using the LMO validation method were above $97\%$. The highest value, equal to $99.9\%$, was achieved for the SVM classifier.

Cohen’s kappa coefficient^50^ $\kappa$ is also computed and listed in Table 1. It checks the pilling grade assessment conformity of two raters: the experts and our classifier. You can see that the changes of $\kappa$ essentially follow the *AC* changes but the $\kappa$ values tend to be lower due to the rating bias caused by the expected agreements of the raters obtained by chance.

Although our study is only a proof-of-concept with a limited textile groups, its implication is that non-subjective texture based classification of OCT images could have practical applications in distinguishing pilling classes. In the standard textile manufacturing process our method will allow for the elimination of time-consuming machine tests as well as biased assessment, which is performed by trained personnel. The main disadvantage of the method is that it requires expensive equipment. However, worldwide research is continuing on the development of OCT instruments with improvements aimed at reducing costs^51^. These devices are also tested for miniaturization^52^. The above modifications may make OCT devices applicable in many industrial areas, including textile industry.

**Table 1:** Quality performance of classifiers expressed as the Accuracy, reliability index $\eta$^49^ and Cohen’s Kappa index $\kappa$. $^1$—method based on Mahalanobis distance, $^2$—method based on centroid distance.

Classifier	Validation	Test T1			Test T2		
		Accuracy (%)	$\eta$ (%)	$\kappa$ (%)	Accuracy (%)	$\eta$ (%)	$\kappa$ (%)
PLS-DA$^1$	LOO	90.2	89.6	0.87	69.9	77.1	0.60
PLS-DA$^2$		79.4	78.9	0.72	65.4	63.2	0.54
PCA-SVM		97.8	98.4	0.97	87.1	91.4	0.83
PCA-LDA		78.4	84.8	0.71	63.9	76.0	0.52
LDA		88.0	91.3	0.84	86.2	91.2	0.82
PLS-DA$^1$	LMO	97.9	98.4	0.97	78.3	86.7	0.71
PLS-DA$^2$		97.7	97.6	0.97	88.3	89.9	0.84
PCA-SVM		99.9	99.9	1.00	77.8	76.1	0.57
PCA-LDA		94.9	97.6	0.93	70.3	86.8	0.60
LDA		98.4	99.3	0.98	90.4	96.1	0.87

## Conclusions

This paper has proposed a computer evaluation method for analyzing both fabric pilling and fuzzing phenomena in the spatial layer above a fabric surface. Using OCT tomography, we obtained spatial images of the pilling layer containing both fuzz and pills. The advantage of our method is that it prevents false pilling ratings, as a result of the detachment of fibers and pills formed by strong and prolonged friction.

The texture of the OCT image was found to provide valuable information about fabric fuzz, which we used to predict pilling by both linear and nonlinear classifiers. The method worked better for the early stage of pilling, when fuzzing predominates and pills have not yet formed. With the nonlinear SVM classifier, accuracy of over $99\%$ was obtained. When an abrasive test stimulating the formation of fabric fuzz is carried out both manually and machine-made, the texture of the pilling layer may not be the same everywhere on the fabric. Aggregation of sample feature components can therefore increase classification accuracy.

## Supplementary Information

Supplementary Information.

## Data Availability

The data sets used and/or analyzed during the current study are available from the corresponding author on reasonable request.
