# Supplementary material for: Prediction of textile pilling resistance using optical coherence tomography
Source: Sci Rep. 2022 Oct 31;12:18341. doi: 10.1038/s41598-022-23230-9 (PMC9622826; doi:10.1038/s41598-022-23230-9)
Supplement: Supplementary file 1 — Supplementary Information. [file 41598_2022_23230_MOESM1_ESM.pdf]

Supplementary material

Prediction of textile pilling resistance using optical coherence tomography

Jarosław Gocławski, Joanna Sekulska-Nalewajko and Ewa Korzeniewska

Table. S1. Tuned SVM parameters for input data components. γ - radial kernel parameter, C - regularization cost parameter, n = 2 ÷ 10 - number of input components.

Test	Params	No. Components (n)								
		2	3	4	5	6	7	8	9	10
T1	C_n	100	10	2	20	10	1	1	1	2
	γ_n	0.2	0.1	1.0	0.5	0.2	0.2	0.2	0.2	0.1
T2	C_n	20	50	10	10	20	10	20	20	5
	γ_n	1.0	0.2	0.2	0.5	0.1	0.1	0.1	0.1	0.1

Table. S2. Summary of the PLS-DA classification for textile OCT images acquired after two abrasion tests based on a 4-class model for textural features. Mahalanobis distance method for measuring the distance to the centroids of the pilling classes was used.

Validation	Group	T1			T2		
		Sensitivity	Specificity	F1	Sensitivity	Specificity	F1
LOO	2	93.4%	98.7%	0.947	69.3%	91.7%	0.725
	3	85.2%	91.6%	0.791	45.5%	84.4%	0.510
	4	82.3%	96.9%	0.864	79.1%	84.1%	0.577
	5	100.0%	100.0%	1.000	94.9%	100.0%	0.974
	OVERALL	**90.2%**	**96.8%**	**0.900**	**72.2%**	**90.0%**	**0.696**
LMO	2	100.0%	99.9%	0.998	59.9%	96.2%	0.721
	3	93.9%	99.3%	0.958	71.2%	87.2%	0.669
	4	97.7%	98.0%	0.958	99.0%	88.1%	0.743
	5	100.0%	100.0%	1.000	100.0%	100.0%	1.000
	OVERALL	**97.9%**	**99.3%**	**0.979**	**82.5%**	**92.9%**	**0.783**

Table. S3. Summary of the PLS-DA classification for textile OCT images acquired after two abrasion tests based on a 4-class model for textural features. Centroid distance method for measuring the distance of the pilling classes was used.

Validation	Group	T1			T2		
		Sensitivity	Specificity	F1	Sensitivity	Specificity	F1
LOO	2	85.8%	94.5%	0.846	56.5%	88.3%	0.614
	3	60.4%	85.8%	0.586	59.4%	86.9%	0.630
	4	71.2%	92.2%	0.741	61.4%	79.5%	0.375
	5	100.0%	100.0%	1.000	80.8%	100.0%	0.894
	OVERALL	**79.4%**	**93.1%**	**0.724**	**64.5%**	**88.7%**	**0.628**
LMO	2	98.6%	99.3%	0.982	89.9%	95.2%	0.875
	3	95.4%	98.5%	0.954	73.7%	97.1%	0.819
	4	96.8%	99.2%	0.972	99.2%	92.6%	0.861
	5	100.0%	100.0%	1.000	96.3%	100.0%	0.981
	OVERALL	**97.7%**	**99.2%**	**0.977**	**89.8%**	**96.2%**	**0.884**

Table. S4. Summary of the PCA-LDA 5 component classification for textile OCT images acquired after two abrasion tests based on a 4-class model for textural features.

Validation	Class	T1			T2		
		Sensitivity	Specificity	F1	Sensitivity	Specificity	F1
LOO	2	80.3%	98.4%	0.872	43.4%	94.5%	0.584
	3	67.1%	86.7%	0.623	55.7%	75.0%	0.290
	4	69.5%	92.6%	0.740	87.5%	84.6%	0.607
	5	100.0%	94.0%	0.894	94.7%	100.0%	0.973
	OVERALL	**79.2%**	**92.9%**	**0.745**	**70.3%**	**88.5%**	**0.613**
LMO	2	86.8%	98.4%	0.929	46.1%	99.9%	0.631
	3	95.4%	86.7%	0.890	21.4%	73.8%	0.012
	4	98.6%	92.6%	0.973	100.0%	94.0%	0.894
	5	100.0%	94.0%	1.000	100.0%	100.0%	1.000
	OVERALL	**95.2%**	**92.9%**	**0.948**	**66.9%**	**91.9%**	**0.634**

Table. S5. Summary of the LDA classification for textile OCT images acquired after two abrasion tests based on a 4-class model for textural features.

Validation	Class	T1			T2		
		Sensitivity	Specificity	F1	Sensitivity	Specificity	F1
LOO	2	83.1%	97.1%	0.872	75.2%	96.9%	0.825
	3	85.4%	92.3%	0.805	76.2%	90.2%	0.729
	4	85.3%	98.0%	0.895	100.0%	94.8%	0.911
	5	100.0%	96.8%	0.948	96.9%	100.0%	0.984
	OVERALL	88.5%	96.1%	0.880	87.1%	95.5%	0.862
LMO	2	94.7%	99.9%	0.971	73.5%	99.8%	0.846
	3	99.4%	98.2%	0.968	96.7%	89.2%	0.770
	4	100.0%	99.9%	0.999	100.0%	99.4%	0.991
	5	100.0%	100.0%	1.000	100.0%	100.0%	1.000
	OVERALL	98.5%	99.5%	0.984	92.5%	97.1%	0.902

Table. S6. Summary of the PCA-SVM classification for textile OCT images acquired after two abrasion tests based on a 4-class model for textural features.

Validation	Class	T1			T2		
		Sensitivity	Specificity	F1	Sensitivity	Specificity	F1
LOO	2	92.4%	99.7%	0.957	72.1%	97.3%	0.811
	3	99.2%	98.8%	0.978	86.1%	89.9%	0.761
	4	100.0%	100.0%	1.000	98.6%	96.0%	0.928
	5	100.0%	98.5%	0.976	96.5%	100.0%	0.982
	OVERALL	97.9%	99.3%	0.978	88.4%	95.8%	0.871
LMO	2	100.0%	100.0%	1.000	69.1%	93.3%	0.746
	3	100.0%	99.9%	0.999	48.8%	86.0%	0.564
	4	99.8%	100.0%	0.999	50.9%	78.4%	0.321
	5	100.0%	100.0%	1.000	100.0%	100.0%	1.000
	OVERALL	100.0%	100.0%	0.999	67.2%	89.4%	0.658

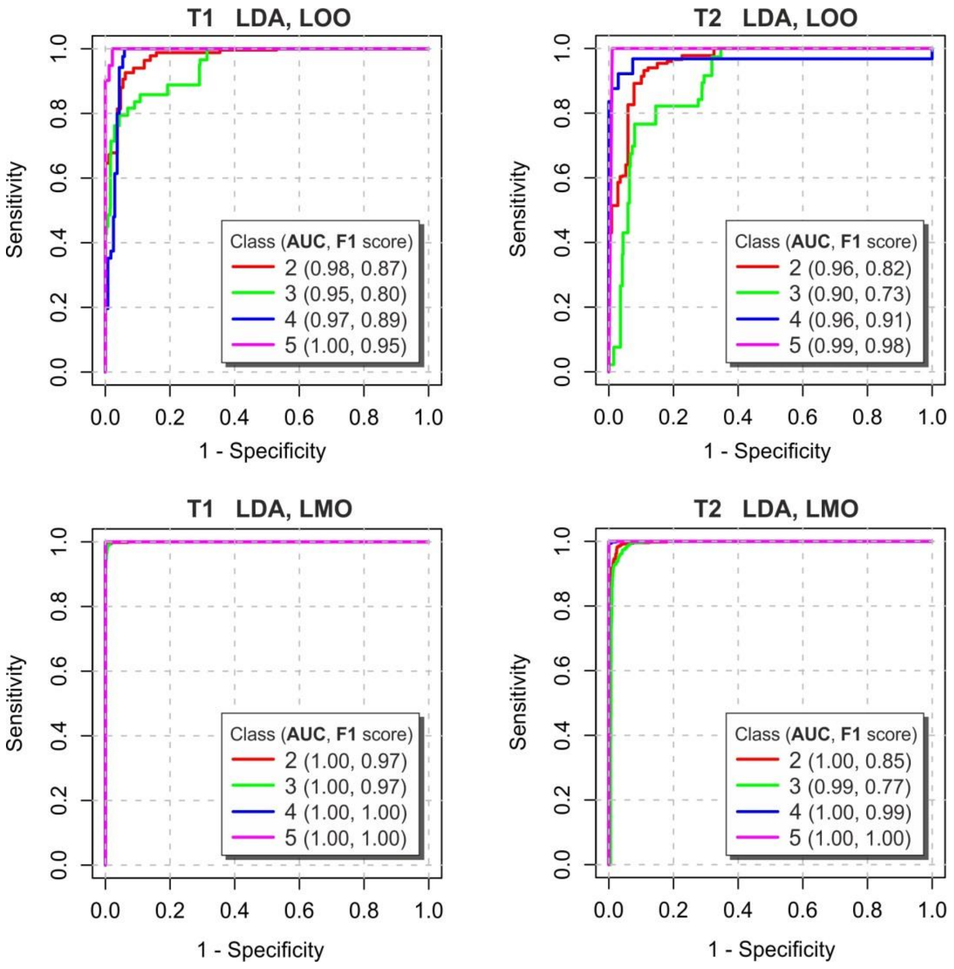

Figure S1. ROC curves for LDA classifier. The area under ROC suggests that the pilling classification based of the texture analysis has a good predictive value for all pilling grades both in the case of T1 and T2 test, when LMO validation technique is applied. AUC values are presented in the Figure legend.

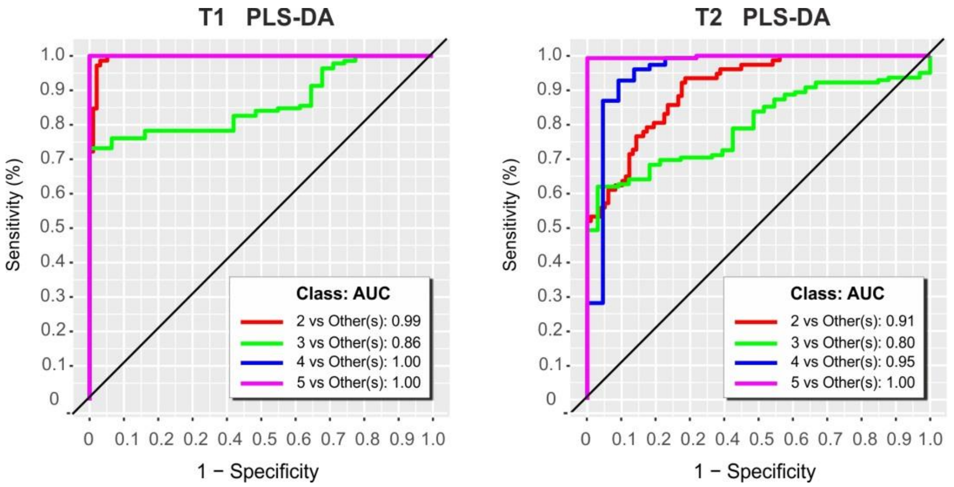

Figure S2. ROC curves for PLS-DA classifier obtained using *mixOmics::auroc* function in R language for a model with 5 components. AUC values are presented in the Figure legend.
